# Survival and inferential analysis in patients with locally advanced breast cancer treated with neoadjuvant chemotherapy and subsequent sentinel lymph node biopsy: prospective single-center study

**DOI:** 10.1186/s41824-024-00202-y

**Published:** 2024-05-22

**Authors:** Johanna Marcela Espejo Niño

**Affiliations:** Nuclear Medicine Department, Hospital Universitario Cruces, Barakaldo, Spain

**Keywords:** Breast cancer, Sentinel node, Neoadjuvant chemotherapy, Survival

## Abstract

**Background:**

The lymph node staging is the major prognostic factor in breast cancer patients. Sentinel lymph node biopsy (SLNB) allows an exactly axillar staging in patients with early disease, but not in locally advance breast cancer (LABC). Our aim was to study, the feasibility and accuracy of the SLNB technique with and without axillar lymphadenectomy (LDN) and with lymph node clipping after neoadjuvant chemotherapy (NAC), in patients with LABC.

**Patients and methods:**

Patients diagnosed with LABC, scheduled for NAC and subsequent surgery and SLNB. Subsequently the patients were scheduled for adjuvant chemotherapy/hormonotherapy and radiotherapy according with the postsurgical results. Main end points were overall survival (OS) disease-free survival (DFS), mortality, SLNB identification rate (IR), sensitivity, false negative rate (FNR) of SLNB versus LDN, negative predictive value (NPV) and overall accuracy.

**Results:**

Our IR with different techniques was between 89.9 and 100%. OS was between 89 and 97%. DFS was between 89.8 and 96.8%. Sensitivity was between 75 and 100%. NPV was between 89.6 and 100%. FNR was between 0 and 25%; and accuracy was between 66 and 72%. We found that survival was lower (*p* < 0.05) in patients with triple negative and Luminal B/HER2 intrinsic subtype; with progression or major partial response in Magnetic Resonance Imaging (MRI) results at the end of NAC and in patients with BRCA1/2 mutation.

**Conclusions:**

Our study presents excellent results of SLNB alone in patients with LABC with complete nodal response with an OS and DFS > 95%. The FNR is very high in partial responders, so we cannot recommend the SLNB alone in LABC. We recommend, in cN+ patients, axillar clipping, SLNB and LDN because in more than 50% of the patients with axillar clipping, this was not found, and because in 36% of the patients with negative LDN, the SLN (Sentinel Lymph Node) obtained was the only positive node, so these techniques together decrease the FNR and improve the node staging, OS and DFS. This study is the first prospective study that assess OS and DFS in patients with LABC, all submitted to SLNB.

## Highlights

1. The sentinel lymph node identification rate in our study was 92.10% that was better than others literature reports.
2. In 36% of the patients with negative LDN, in the group of axillar clip plus SLNB and LDN, the sentinel lymph node was the only positive node.
3. From the patients with positive clinical nodal staging (cN+), 51.2% presented complete nodal response.
4. 65.62% of the patients with complete radiological response presented complete pathological response.
5. The patients with axillar clipping plus SLNB and additional positive LDN did not present false negative results, that means that we obtain a full coincidence between a positive SLNB and positive LDN.
6. In 11 patients the clipping node was not found (45.8%) which means that in almost half of the patients the clip did not improve axillary staging.
7. Mortality rate was 7%.

## Introduction

The safety of Sentinel lymph node biopsy (SLNB) without axillar lymphadenectomy (LDN) in patients diagnosed with early-stage breast cancer has been well established in the NSABP-B32 (Krag et al. 2004), IBSGC-23-01 (Galimberti et al. 2018), AATRM-048 (Solá et al. 2013) and ACOSOG-Z0011 (Giuliano et al. 2017) studies. The use of SLNB without LDN after neoadjuvant chemotherapy (NAC) in patients diagnosed with locally advance breast cancer (LABC) continues being controversial and in general, there is still no consensus on its use in patients with these characteristics. For this reason, radical axillary LDN remains the standard of care in most centers. The high False Negative Rate (FNR) of SLNB, in this scenario, limit the use of this technique and currently, there is a few literature about the effects on survival of patients with LABC treated with SLNB.

It has been postulated that tumor cells alter the lymphatic vessels in their colonization, and it has been postulated that chemotherapy finishes altering the lymphatic vessels too, producing tumor death and fibrosis. In these cases, the drainage of the sentinel lymph node would be altered, but not the drainage of the non-sentinel nodes (Mocellin et al. 2016), and tumoral cells would remain, which would increase the false negative results.

In the SENTINA study (Kuehn et al. 2013) the Identification Rate (IR) was 80.1% and the FNR was 14.2%. The ACOSOG- Z1071 study (Boughey et al. 2013) reported high FNR (12.6%) too, even when 2 or more sentinel nodes were obtained, which exceeded their pre-set threshold of 10% and found that, the use of a clip in the positive node, reduces the FNR to 6.8% (Boughey et al. 2013). The authors concluded that a different treatment or approach should be performed in these patients. In the SENTINA study, there were two factors that improved IR and accuracy: the dual method of sentinel node mapping (using radiotracer and blue dye) and obtaining 2 or more sentinel nodes (Kuehn et al. 2013).

For all this reasons, we have developed a prospective inferential study to assess our IR, mortality, FNR and the survival of the patients with LABC treated with NAC who underwent to SLNB.

## Patients and methods

### Type of study

Single-center prospective study, between July 2013 and July 2023, performed at Hospital Universitario Cruces.

### Inclusion and exclusion criteria

The patients included were diagnosed with LABC, scheduled for NAC and subsequent surgery and SLNB; with clinical staging, following the American Joint Committee on Cancer** (**AJCC) 8th edition TNM criteria (T: Tumor, N: Nodes; M: Metastasis) (Amin et al. 2017) for diagnosis and response; and radiological staging with mammography, ultrasonography and/or subsequent Magnetic Resonance Imaging (MRI); with pathological study including histological type, prognostic markers, intrinsic subtype and when suspected lymph node positivity in axilla, verification by pathology with core needle biopsy. Subsequently the patients were scheduled for adjuvant chemotherapy/hormonotherapy and radiotherapy (RT), according with the postsurgical results. Genetic tests were also performed, searching for the penetrance genes BRCA1 and BRCA2, following the criteria of the Spanish Society of Medical Oncology (SEOM in spanish). The clinical criteria for high risk of hereditary/familial breast cancer were: A case of cancer less than or equal to 40 years. Diagnosis of breast and ovarian cancer in the same individual. Two or more cases of breast cancer, one of which is under 50 years of age or bilateral. One case of breast cancer less than or equal to 50 years old or bilateral and one case of ovarian cancer in 1st or 2nd degree familial. Three cases of breast and ovarian cancer (at least one ovarian case) in 1st or 2nd degree relatives. Two cases of ovarian cancer in 1st and 2nd degree relatives. A case of breast cancer in a male and a 1st or 2nd degree relative with breast or ovarian cancer. Moderate risk criteria include: Two 1st degree relatives if both have been diagnosed between the ages of 51 and 60. 1 relative in the 1st and 2nd degree (mother or sister and maternal aunt or grandmother), if the sum of their ages is less than or equal to 118 years (González-Santiago et al. 2019). Informed consent was obtained from all individual participants included in the study.

The patients excluded were those diagnosed with inflammatory cancer and who did not undergo to SLNB.

Therapeutic protocols were scheduled on an individualized basis, according to the histological and molecular characteristics of the tumor.

### Pathological and radiological criteria

The study used the Molecular Classification of Breast Cancer 2020 definitions by Immunohistochemical (IHC) phenotype (Tsang and Tse 2020): Luminal A-like (positive Estrogen Receptor *«*ER*»:* ER+; Progesterone Receptor *«*PR*»* ≥ 20%: PR ≥ 20%; negative Human Epidermal Growth Factor Receptor *«*HER”*»:* HER2−, Ki67 < 20%); Luminal B-like (ER+, PR < 20% and/or HER2+ and/or Ki67 ≥ 20%); HER2-overexpression (ER−, PR−, HER2+) and basal-like (triple negative: ER−, PR−, HER2−).

Tumor and lymph node response was evaluated first by MRI and then with pathology results.

The study used, for radiological response, the International Union for Cancer Control (Wittekind et al. 2005) criteria, that distinguishes 4 types of local radiological response:

1. Non-response: no change in tumor size.
2. Minor partial response (mPR): tumor size decreases less than 50% of the greater diameter.
3. Major partial response (MPR): tumor size decreases by more than 50% of the greater diameter.
4. Complete response (CR): disappearance of the lesions and absence of areas of pathological enhancement.

*SLNB protocol* The day before surgery, all patients underwent a lymphoscintigraphy of the breast with tumor involvement, in search of the sentinel lymph node. In the protocol, 111 MBq of [Tc-99m]Tc-Nanocolloid in a 0.2 mL injection, was performed subdermally and periareolarly, in the breast quadrant where the tumor was initially located. 1–2 h later, planar scintigraphic images of 180 s each were performed in anterior, lateral and anterior oblique projections of the affected side and subsequently, the sentinel lymph node observed in the oblique image was marked on the skin with indelible ink. Single-photon emission computed tomography (SPECT) with low-dose computed axial tomography (CT) was performed if localization of the sentinel lymph node was not possible and in cases with an axillary clip.

On the day of surgery and after anesthesia, subdermal injections with blue dye (methylene blue, 2 mL) were performed in the four quadrants of the affected breast. During the surgical procedure, the sentinel lymph node with the highest activity was detected with the gamma detector probe (EUROPROBE 3.2, Eurorad) and extracted for pathological study; Likewise, lymph nodes with an activity greater than 10% of the maximum uptake of the sentinel lymph node were extracted and the nodes stained with blue dye and that were visually suspicious for the surgeon (Buscombe et al. 2007). If the clip node was not the sentinel node, pathology looked for the clip node in the LDN tissue.

If there was no migration of the radiotracer to any sentinel lymph node, a second subdermal and periareolar injection of 37 MBq Tc-99m]Tc-Nanocolloid in a 0.1 mL injection was performed, in the same tumor quadrant, and after 30 min the images were repeated: planar scintigraphic images and SPECT/low-dose CT for localization. If there was no migration with re-injection, the surgical team was immediately notified and the sentinel lymph node was searched on the day of surgery, first with planar images and then in the surgical bed with the gamma detector probe.

The sentinel lymph node was studied using the One Step Nucleic Acid Amplification OSNA method, which quantifies the expression of cytokeratin 19 (CK19), as a tumor cell marker, in the mRNA of the sentinel lymph node (Tsujimoto et al. 2007). The cutoff value of 2.5 × 102 CK19 mRNA copies/μL represents the upper limit of copy number in histopathologically negative lymph nodes. The cutoff value for micrometastasis was between 250 and 5000 copies of CK19 mRNA/μL and the cutoff value for macrometastasis was more than 5000 copies of CK19 mRNA/μL (Terrenato et al. 2017). Lymph nodes in patients without CK19 expression were studied by immunohistochemistry (IHC).

In our center, we have a breast committee made up of medical and surgical specialists who agree on the diagnosis, prognosis and therapy criteria for all breast cancer patients following the main European guidelines as they are updated. Since 2017, when a suspicious lymph node is clinically detected, staging is carried out with core needle biopsy, performed by the radiodiagnostic team, seeking verification of positivity of malignant cells and then, a clip is placed in the biopsied lymph node. During surgery, the lymph node with a clip is searched in the SLNB and the LDN bed.

*Data analysis* First, a general analysis was performed on all patients who met the inclusion criteria. Subsequently, an analysis was carried out in four groups: the first was the SLNB and LDN group, which correspond to the patients studied between 2013 and 2017, before the ACOSOG Z0011 study. Then we implemented the axillary clip technique with SLNB and LDN, that was the second group. In the third group were the patients with CR in post-NAC MRI who underwent SLNB without LDN and the fourth was the axillary clip group with SLNB without LDN.In the inferential analysis, overall survival (OS), disease-free survival (DFS), mortality, SLNB identification rate (IR), sensitivity, false negative rate (FNR) of SLNB versus LDN, negative predictive value (NPV) and overall accuracy were obtained as well as the Kaplan Meier curves, using the SPSS statistical package version 29.0.1.0. For continuous variables, the mean and standard deviation were calculated.

We defined the IR like the number of patients who had successfully identified sentinel lymph nodes divided by the total number of women in whom a SLNB was attempted. Four formulas of test performance were made: sensitivity = [true positives/ (true positives + false negatives)], FNR = [false negatives/ (true positives + false negative)], NPV = [true negatives/ (true negatives + false negatives)], and overall accuracy [(true positives + true negatives)/total number of successful SLNB].

## Results

### General analysis

Since 2013 to 2023, 114 patients met the inclusion criteria, with mean age of 53.16 years (Standard Deviation: SD ± 10.31); of which 87.8% corresponded to patients with clinical tumor staging (cT) cT2–T4 and 73.7% with clinical nodal staging (cN) cN1–N3. 89.5% of the patients had infiltrating ductal carcinoma (IDC). 38.6% were grade II and 57.8% were grade III. 26.3% were of the triple-negative intrinsic subtype; 26.3% were Luminal/HER2 and 18.4% were HER2 (Table 1).

**Table 1:** Histopathological characteristics, therapeutic protocol and post-NAC MRI and pathology results

	Total	SLNB and LDN	SLNB, CLIP and LDN	SLNB alone	SLNB and CLIP
	n = 114 (%)	n = 59 (%)	n = 24 (%)	n = 26 (%)	n = 5 (%)
Tumor					
cT1	14 (12.3)	7 (11.9)	6 (25)		1 (20)
cT2	76 (66.7)	39 (66.1)	11 (45.8)	22 (84.6)	4 (80)
cT3	19 (16.7)	11 (18.6)	6 (25)	2 (7.7)	
cT4	5 (4.4)	2 (3.4)	1 (4.2)	2 (7.7)	
Nodes					
cN0	30 (26.3)	15 (25.4)		14 (53.8)	1 (20)
cN1	77 (67.5)	38 (64.4)	23 (95.8)	12 (46.2)	4 (80)
cN2	6 (5.3)	5 (8.5)	1 (4.2)		
cN3	1 (0.9)	1 (1.7)			
Histological type					
IDC	102 (89.5)	50 (84.7)	22 (91.7)	25 (96.2)	5 (100)
DCIS	2 (1.8)	2 (3.4)			
ILC	7 (6.1)	4 (6.8)	2 (8.3)	1 (3.8)	
Tubular	1 (0.9)	1 (1.7)			
Medullar	1 (0.9)	1 (1.7)			
Metaplastic	1 (0.9)	1 (1.7)			
Grade					
I	4 (3.45)	2 (3.4)	1 (4.2)	1 (3.8)	
II	44 (38.6)	21 (35.6)	13 (54.2)	7 (26.9)	3 (60)
III	66 (57.9)	36 (61)	10 (41.7)	18 (69.2)	2 (40)
Intrinsic subtype					
Luminal A	8 (7)	4 (6.8)	2 (8.3)	1 (3.8)	1 (20)
Luminal B	25 (21.9)	14 (23.7)	10 (41.7)	1 (3.8)	
Luminal B/HER2	30 (26.3)	19 (32.2)	4 (16.7)	6 (23.1)	1 (20)
HER2	21 (18.4)	9 (15.3)	4 (16.7)	8 (30.8)	
Triple Negativo	30 (26.3)	13 (22)	4 (16.7)	10 (38.5)	3 (60)
Radiological response					
MPR	51 (44.7)	26 (44.1)	10 (41.7)	11 (42.3)	4 (80)
mPR	22 (19.3)	12 (20.3)	8 (33.3)	2 (7.7)	
CR	32 (28.1)	15 (25.4)	5 (20.8)	11 (42.3)	1 (20)
Progression	1 (0.9)	1 (1.7)	1 (4.2)	2 (7.7)	
Non-response	8 (7)	5 (8.5)			
Tumoral response					
ypT0	48 (42.5)	25 (41.4)	7 (29.2)	14 (53.8)	2 (40)
ypT1	50 (44.2)	24 (40.7)	14 (58.3)	9 (34.6)	3 (60)
ypT2	12 (10.6)	8 (13.6)	2 (8.3)	2 (7.7)	
ypT3	2 (1.8)	1 (1.7)	1 (4.2)	1 (3.8)	
ypTis	1 (0.9)	1 (1.7)			
Not valid**	1* (0.9)				
Nodal response					
ypN0	66 (58.8)	25 (42.4)	11 (45.8)	26 (100)	5 (100)
ypN1	31 (27.2)	23 (39)	8 (33.3)		
ypN2	16 (13.2)	10 (16.9)	5 (20.8)		
ypN3	1 (0.9)	1 (1.7)			
LDN	83 (72.8)	59 (100)	24(100)		
LDN positive					
SLNB+	16 (19.3)	9 (15.3)	7 (29.2)		
SLNB−	3 (3.6)	3 (5.1)			
Non-migration	5 (6.02)	4 (6.8)	1 (4.2)		
LDN negative					
SLNB+	21 (25.3)	15 (25.4)	6 (25)		
SLNB−	34 (40.9)	26 (44.1)	8 (33.3)		
Non-migration	4 (4.8)	2 (3.4)	2 (8.3)		
SLNB without LDN	31 (27.2)			26 (100)	5 (100)
SLNB+					
SLNB−	31 (100)			26 (100)	5 (100)
Non-migration					
Therapeutic protocols					
Trastuzumab/Pertuzumab/AC/ Docetaxel	38 (33.3)	18 (30.6)	8 (33.3)	11 (42.3)	1 (20)
TAC	36 (31.5)	19 (32.2)	13 (54.2)	3 (11.5)	1 (20)
CBCDA / Docetaxel / AC	24 (21.0)	10 (16.9)	2 (8.3)	9 (34.6)	3 (60)
Trastuzumab / AC / Docetaxel	13 (11.4)	11 (18.6)		2 (7.7)	
CBDCA /Trastuzumab / Pertuzumab / AC / Paclitaxel	2 (1.7)		1 (4.2)	1 (3.8)	
Letrozol	1* (0.8)	1 (1.69)			

IDC: infiltrating ductal carcinoma. DCIS: ductal carcinoma in situ. ILC: infiltrating lobular carcinoma. HER2: human epidermal growth factor receptor. TAC: Docetaxel, Adriamycin and Cyclophosphamide. AC: Adriamycin and Cyclophosphamide. CBDCA: Carboplatin. (*) Patient with cardiac, renal and pulmonary comorbidities. CR: Complete response, MPR: Major partial response, mPR: Minor partial response. ypT: tumor response to NAC, ypN: lymph node response to NAC. (**) technical failure in sample processing. LDN: Lymphadenectomy. SLNB(+): SLNB positive, SLNB (−): SLNB negative

Therapeutic protocols included hormone therapy, chemotherapy, and immunotherapy (Table 1). The most frequents treatments were Trastuzumab/Pertuzumab/AC (Adriamycin, and Cyclophosphamide)/Docetaxel and TAC (Docetaxel, Adriamycin, and Cyclophosphamide).

In the post-QTNA MRI results, 44.7% of the patients had CR, 28.1% had MPR, 19.3% had mPR and 7% had stability.

All patients underwent SLNB, (Table 1), the mean number of sentinel nodes removed was 2.13, (SD ± 1.2). From the 114 patients, 6 SLNB were analyzed by immunohistochemistry (IHC), because they were CK19 negative. The other 108 patients the SLNB were analyzed by OSNA method.

58.8% (n = 67) of the patients were SLNB negative (SLNB−), 33.3% (n = 38) were SLNB positive (SLNB+); 7.9% (n = 9) had non-migration of the radiotracer in the SLNB. That means that our IR was 92.10%. Only 72.8% (n = 83) of the patients underwent to LDN. The mean number of nodes removed in LDN was 10.25 (SD ± 7.3).

Pathological and MRI post-NAC results of all 114 patients studied (Table 1) showed that from the 32 patients with CR in MRI post-NAC, 21 (65.62%) were ypT0 ypN0 in the pathological results too. From the 73 patients with partial response (PR), in MRI post-NAC, 16 were finally ypT0ypN0 by pathology and from the patients with positive clinical nodal staging (cN+; n = 84), 51.19% (n = 43) became ypN0, by pathological results, (Table 1).

86 patients were subsequently treated with adjuvant treatment (chemotherapy and/or hormone therapy). 28 patients were not treated with adjuvant and belong to the intrinsic triple negative subtype. From these patients without adjuvant treatment, 5 died.

16 patients were not treated with adjuvant RT; of these 16 patients, 2 died: 1 patient was LuminalB/HER2 with PR in MRI post-NAC results, SLNB(−) and ypT1ypN0 and 1 was patient triple negative with PR, in MRI post-NAC results, SLNB(−) and ypT2ypN0.

From the 114 patients, 8 (7.01%) died: 5 patients were triple-negative intrinsic subtype, 2 were Luminal B/HER2 and 1 was Luminal B. 3 patients had BRCA1/2 mutation, 1 of them was a non-migration of the radiotracer, with positive LDN (18+/22). 5 patients were ypT1-2, and 2 patients were ypT0 ypN0. One patient had no final ypT classification due to technical failures in sample processing.

Seven patients with LDN and 1 without LDN, that was SLNB(−) ypT0ypN0 with CR in MRI post-NAC results, who died due to progression of contralateral locally advanced cancer.

**Fig. 1:**
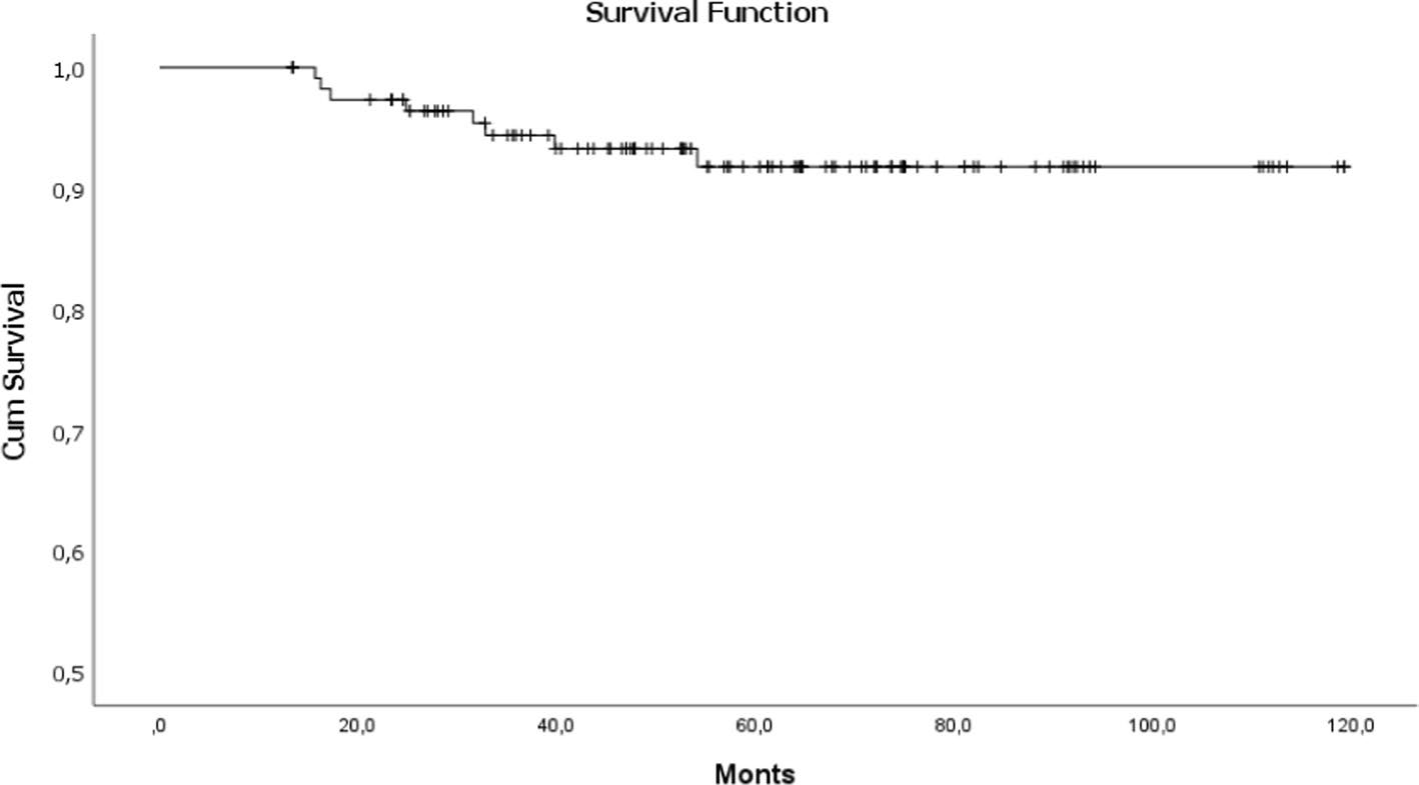
Overall survival function. From the 114 patients, 8 (7.01%) had die. In the ten years of our study (120 months), the probability of survival of the 114 patients was 112.086 months, (95% confidence interval 106.2–116.9), with an OS of 92% and a DFS of 93%

In the ten years of our study (120 months), the probability of survival of the 114 patients was 112,086 months, (95% confidence interval «CI»: 106.2–116.9), with an OS of 92% (Fig. 1) and a DFS of 93%.

### ***Group of patients who underwent to SLNB and LDN, (***Table 1***):***

From the 83 patients with LDN, 59 patients were submitted to LDN and SLNB; 88% were T2–T4, 74.6% were cN+, 84.7% were IDC, 61% were grade III and 32% were Luminal B/HER2.

The MRI and pathological post-NAC results (Table 1) showed that 44% of the patients were MPR and 25% of the patients were CR in post-NAC MRI. 42% of the patients were ypT0ypN0.

From the 43 negative LDN, 15 were SLNB(+), that means that, the SLN was the only positive node in 25.4% of the patients who underwent to LDN in this group (Table 1).

Our IR was, with this technique, 89.8% and sensitivity, VPN, FNR and accuracy were: 75%; 89.6%; 25% and 66% respectively.

The survival probability for patients who underwent to SLNB and LDN (n = 59), with a follow-up of 120 months, was 110,93 months (SD ± 3.7; 95% CI 104.90–116.8) with an OS of 90.3% with a DFS of 91.6%.

### ***Group of patients who underwent to axillar clipping, SLNB and LDN, (***Table 1***)***

From the 83 patients with LDN, 24 were submitted previously to an axillar clipping technique. 46% of the patients were cT2, 96% of the patients were cN1, the most frequent histological subtype was IDC (91%), and the most frequent intrinsic subtype was Luminal B (41%).

The MRI and pathological post-NAC results (Table 1) showed that 42% of the patients were MPR and 33% of the patients were mPR in post-NAC MRI. 29% of the patients were ypT0ypN0.

From the 24 patients with axillar clipping, SLNB and LDN, 9 were positive LDN (37.5%) and 16 were negative LDN (66.6%).

From these patients, 14 were SLNB(+), 3 were non-migrations and 8 were SLNB (−), (Table 1).

From the 16 patients with negative LDN, 6 were SLNB(+), that means that, the sentinel node was the only positive node in 37.5% of the patients who underwent to LDN in this group.

From the 8 patients with positive LDN, there were not SLNB(−). In this case there were no false negative results. In 8 patients the clipping node was the sentinel node. In 3 patients the clipping node was different to the sentinel node, and in 13 patients the clipping node was not found.

Our IR, with this technique, was 91.6% and sensitivity, VPN, FNR and accuracy were 100%; 100%; 0% and 72% respectively.

**Fig. 2:**
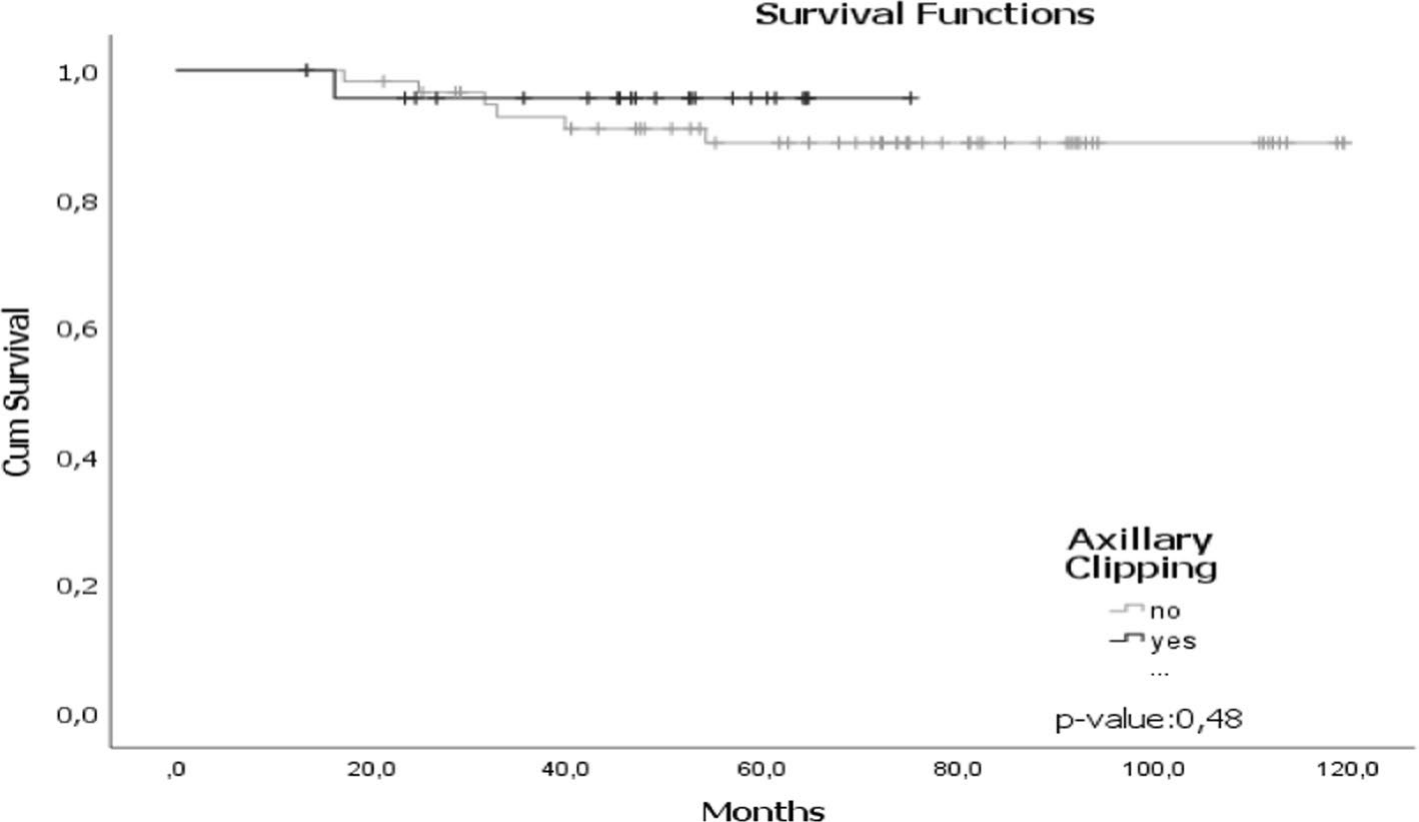
Survival function for LDN and SLNB. In our series, the survival probability for patients who underwent to SLNB and LDN (n = 59), with a follow-up of 120 months, was 110.93 months (SD ± 3.7; 95% confidence interval 104.90–116.8) with an OS of 90.3%, with a DFS of 91.6%

The survival probability for patients who underwent to axillar clipping plus SLNB plus LDN (n = 24), with a follow-up of 80 months, was 72.53 months (SD ± 2.5; 95% CI 67.6–77.43) with an OS of 96% (Fig. 2) with a DFS of 95.8%.

### ***Group of patients who underwent to SLNB alone (***Table 1***)***

From the patients who underwent to SLNB alone, 84.6%, (n = 26), were T2; 53.8% were cN0, 96.2% were IDC, 69.2% were grade III and 38.5% were triple negative.

The MRI and pathological post-NAC results (Table 1) showed that 42.3% patients were radiological CR and 42.3% were MPR in the tumor but with axillar response.

In these group, 100% were SLNB(−). 100% of patients migrated and 100% were ypN0 post-NAC.

Our IR was, with this technique, 100%.

The survival probability for patients who underwent to SLNB alone (n = 26), with a follow-up of 93 months, was 87.23 months (SD ± 2.3; 95% CI 82.63–91.83) with an OS of 97% with a DFS of 96.8%.

**Fig. 3:**
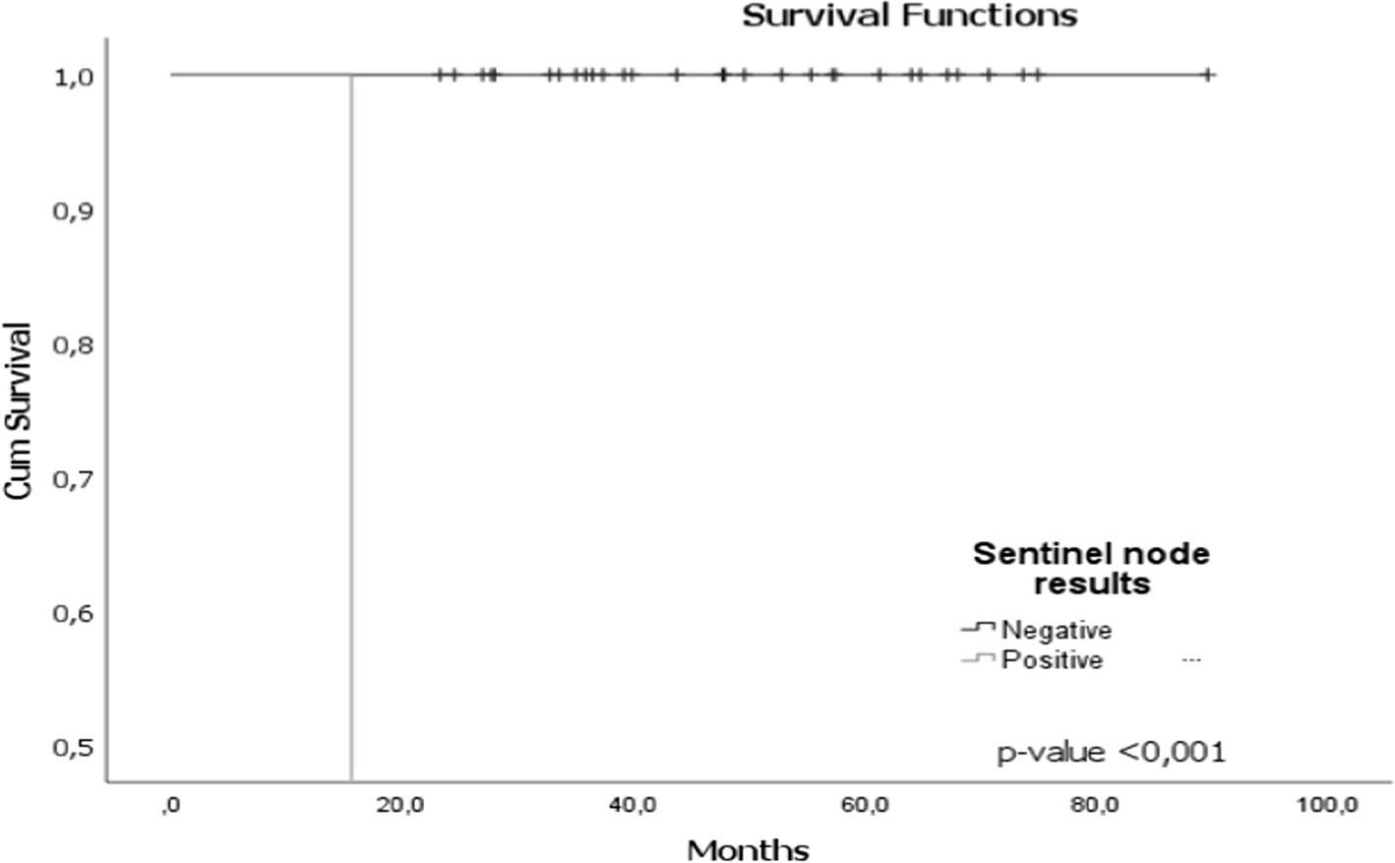
Survival function with respect to the outcome of SLNB. The survival probability for patients who underwent to SLNB alone with a follow-up of 93 months, was 87.23 months, with an OS of 97%, with a DFS of 96.8%. The patient who died was cT2cN0, triple negative intrinsic subtype, with SLNB-and ypT0ypN0, but posteriorly, the patient presented IDC in the contralateral breast that progressed. 1 patient was IDC, HER2 and presented pulmonary, bone and adrenal relapse and still survives

The patient who died was cT2cN0, triple negative intrinsic subtype, with SLNB(−) and ypT0ypN0, but posteriorly, the patient presented IDC, triple negative intrinsic subtype in the contralateral breast that progressed (mentioned in the general analysis). 1 patient was IDC, HER2 and presented pulmonary, bone and adrenal relapse and still survives (Fig. 3).

### ***Group of patients who underwent to axillar clipping and SLNB alone (***Table 1***):***

There were 5 patients who underwent to axillar clipping and SLNB alone. 80% were T2; 80% were cN1, 100% were IDC, 60% were grade II and 60% were triple negative.

MRI and pathologic post-NAC results of patients with axillar clipping and SLNB alone showed that 80% patients were MPR in the tumor but with axillar response, and 20% were CR. 100% were ypN0.

The 5 patients were negative SLNB. In 3 patients the sentinel node and the clipping node was the same. In the other 2 patients the clipping node was not found.

Our IR was 100%. All these patients have survived.

### Inferential statistics

**Fig. 4:**
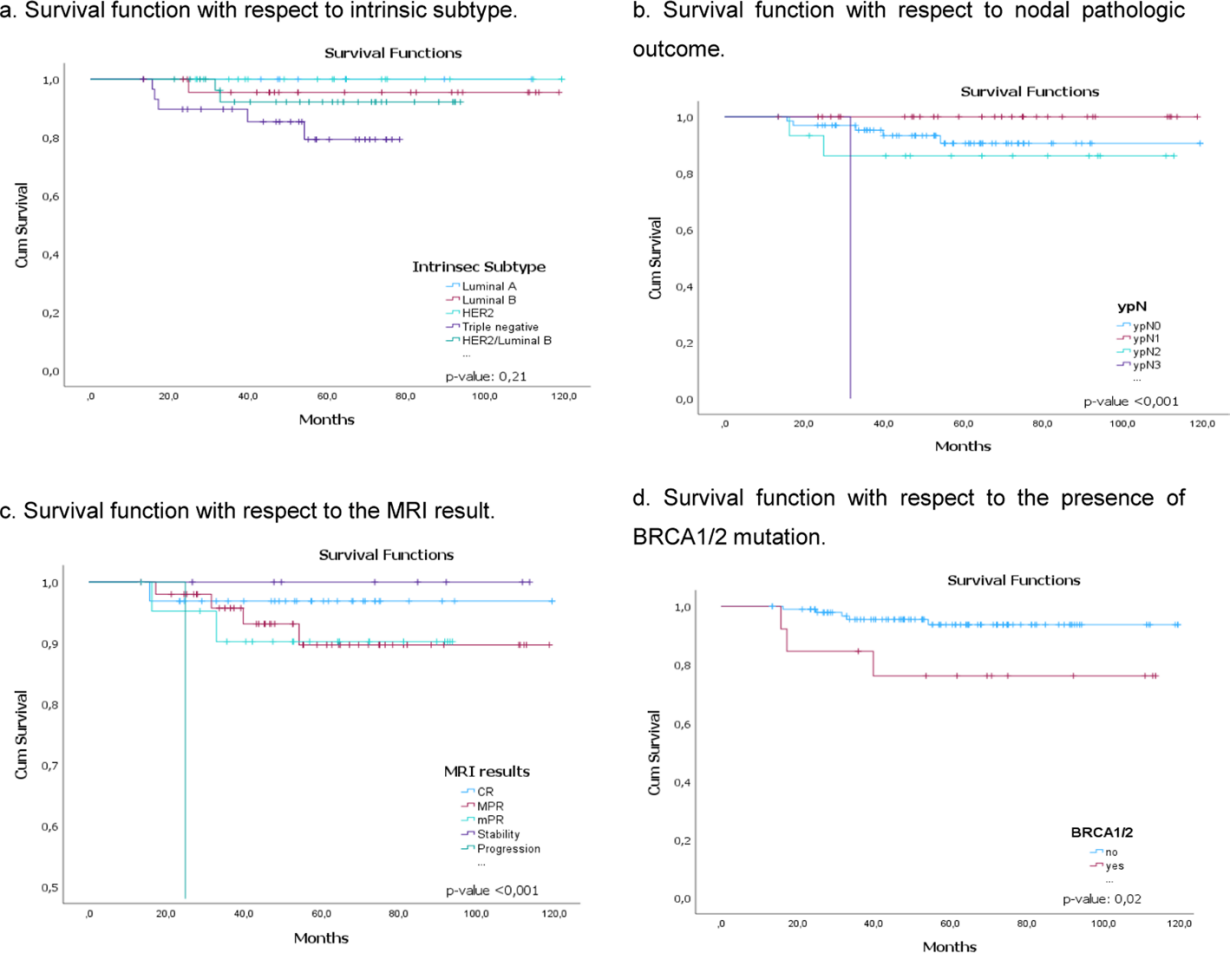
Survival function for SLNB. **a** Survival function with respect to intrinsic subtype. **b** Survival function with respect to nodal pathologic outcome. **c** Survival function with respect to the MRI result. **d** Survival function with respect to the presence of BRCA1/2 mutation. In our series, the survival probability in the 114 patients was lower for patients with **a** triple negative and Luminal B/HER2 intrinsic subtype; **b** pathological node response ypN2-3, with **c** progression or MPR in post-NAC MRI results and **d** in patients presenting BRCA1/2 mutation

We found, with statistical significance (*p* < 0.05), that survival in the 114 patients was lower for those with triple negative and LuminalB/HER2 intrinsic subtype; ypN2-3 pathological node response, with progression or MPR in MRI post-NAC results and in patients with BRCA1/2 mutation, (Fig. 4a–d respectively).

**Fig. 5:**
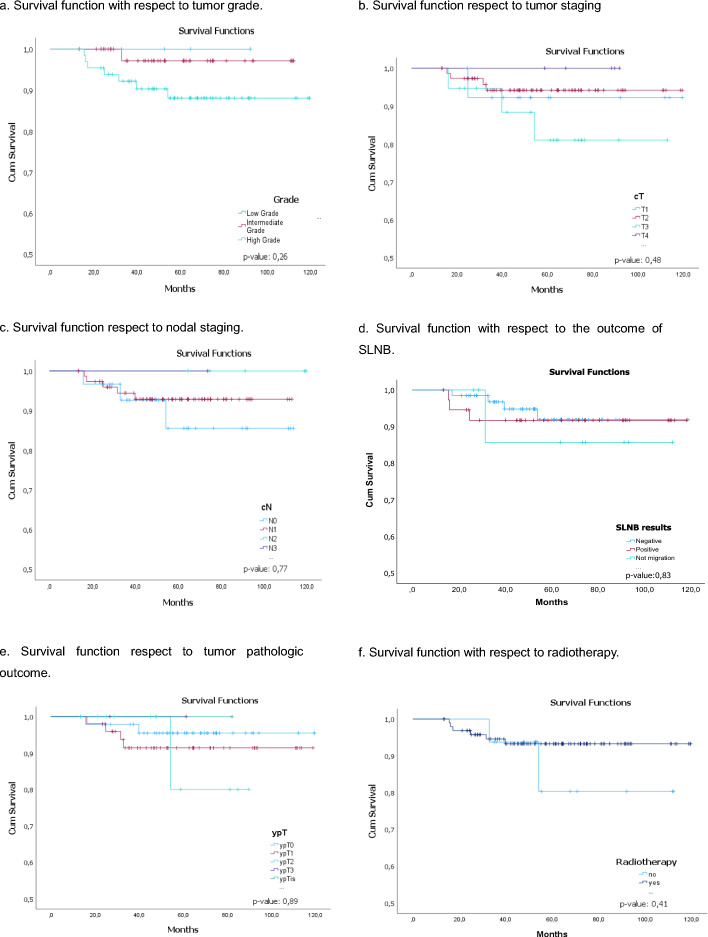
Survival function for SLNB. **a** Survival function with respect to tumor grade. **b** Survival function respect to tumor staging. **c** Survival function respect to nodal staging. **d** Survival function with respect to the outcome of SLNB. **e** Survival function respect to tumor pathologic outcome. *f*. Survival function with respect to radiotherapy. With *p* > 0.05 the survival decreases in patients with **a** high degree of tumor differentiation, **b** in patients cT3, **c** in patients cN0, **d** patients who did not have SLNB migration, **e** ypT2-3 tumoral pathology response and **f** who were not treated with radiotherapy

Although, with a *p* > 0.05, but with a clear tendency, the survival decreases in patients with a high degree of tumor differentiation, in patients cT3, cN0, patients who did not have SLNB migration, ypT2–3 tumoral pathology response and who were not treated with RT, (Fig. 5a–f, respectively).

There is a statistically significant difference (*p* = 0.001) between the result of SLNB and the initial nodal staging (cN). 73.7% of patients with SLNB( +) were cN1. 61.2% of the patients with SLNB(−) were cN1. 88.9% of the non-migrations were cN1, (Table 2).

**Table 2:** SLNB results with respect to initial nodal staging, intrinsic subtype, tumor response, nodal response, MRI response and LDN results

	Overall	Negative	Positive	Non-migration	*p* value
	*n* = *114*	*n* = *67*	*n* = *38*	*n* = *9*	
cN, n (%)					*0.001*
N0	30 (26.3%)	24 (35.8%)	6 (15.8%)	0 (0.00%)	
N1	77 (67.5%)	41 (61.2%)	28 (73.7%)	8 (88.9%)	
N2	6 (5.3%)	2 (3.0%)	4 (10.5%)	0 (0.00%)	
N3	1 (0.9%)	0 (0.00%)	0 (0.00%)	1 (11.1%)	
I.S. n (%)					*0.003*
Luminal A	8 (7.0%)	4 (6.0%)	4 (10.5%)	0 (0.00%)	
Luminal B	25 (21.9%)	8 (11.1%)	16 (42.1%)	1 (11.9%)	
HER2	21 (18.4%)	13 (19.4%)	5 (13.2%)	3 (33.3%)	
Triple negative	30 (26.3%)	23 (34.3%)	7 (18.4%)	0 (0.00%)	
HER2/Luminal B	30 (26.3%)	19 (28.4%)	6 (15.8%)	5 (55.6%)	
ypT, n (%)					*0.017*
ypT0	48 (42.5%)	37 (56.1%)	9 (23.7%)	2 (22.2%)	
ypT1	50 (44.2%)	22 (33.3%)	23 (60.5%)	5 (55.6%)	
ypT2	12 (10.6%)	5 (7.6%)	6 (15.8%)	1 (11.1%)	
ypT3	2 (1.8%)	1 (1.5%)	0 (0.00%)	1 (11.1%)	
ypN, n (%)					< *0.001*
ypN0	67 (58.7%)	61 (91.0%)	2 (5.3%)	4 (44.4%)	
ypN1	31 (27.2%)	3 (4.5%)	26 (68.4%)	2 (22.2%)	
ypN2	15 (13.2%)	3 (4.5%)	10 (26.3%)	2 (22.2%)	
ypN3	1 (0.9%)	0 (0.00%)	0 (0.00%)	1 (11.1%)	
Final MRI results N (%):					*0.02*
CR	32 (28.1%)	24 (35.8%)	7 (18.4%)	1 (11.1%)	
MPR	51 (44.7%)	33 (49.3%)	16 (42.1%)	2 (22.2%)	
mPR	22 (19.3%)	7 (10.4%)	11 (28.9%)	4 (44.4%)	
Non-response	8 (7.0%)	3 (4.5%)	3 (7.9%)	2 (22.2%)	
Progression	1 (0.9%)	0 (0.00%)	1 (2.6%)	0 (0.00%)	

	Overall	Negative	Positive	Non-migration	*p* value
	*n* = *83*	*n* = *37*	*n* = *37*	*n* = *9*	
LDN, n (%)					*0.020*
Negative	58 (69.9%)	33 (89.1%)	21 (36.2%)	4 (44.4%)	
Positive	25 (30.1%)	4 (10.9%)	16 (64.0%)	5 (55.6%)	

73.7% of patients with SLNB(+) were initially cN1. 61.2% with SLNB(−) were initially cN1. 88.9% of non-migrations were initially cN1. 42.1% of patients with SLNB(+) were initially of the Luminal B intrinsic subtype. 34.3% with SLNB(−) were initially triple negative. 55.6% of patients with non-migration were Her2/Luminal B initially. 56.1% of SLNB(−) patients were finally ypT0. 60% of SLNB(+) patients were finally ypT1. 55.6% of non-migrations were finally ypT1. 91% of patients with SLNB(−) were finally ypN0. 68.4% of SLNB(+) were finally ypN1. 44.4% of non-migrations were finally ypN0. 49.3% of SLNB(−) patients present MPR on post-NAC MRI. 42.1% of SLNB (+) patients had MPR. 44.4% of non-migrations present mPR. negative in the LDN. 64% of SLNB (+) patients were positive in the LDN. 89% of the SLNB(−) patients were LDN(−). I. S. Intrinsic subtype. LDN: Lymphadenectomy SLNB: sentinel node biopsy, ypT: tumor staging after NAC. RC: Complete response, MPR: Major partial response, mPR: Minor partial response

There is a statistically significant difference, (*p* = 0.003), between SLNB result and intrinsic subtype. 42.1% of the patients SLNB(+) were Luminal B. 34.3% of patients SLNB(−) were triple negative. 55.6% of patients with non-migrations were Her2/Luminal B, (Table 2).

There is a statistically significant difference, (*p* = 0.017), between the SLNB result and the tumoral response. 56.1% of the patients SLNB(−) were finally ypT0. 60% of patients SLNB(+) were finally ypT1. 55.6% of the non-migrations were finally ypT1, (Table 2).

There is a statistically significant difference, (*p* < 0.001), between the SLNB result and the final ypN0 result. 91% of patients SLNB(−) were finally ypN0. 68.4% of the patients SLNB(+) were finally ypN1. 44.4% of the non-migrations were finally ypN0, (Table 2).

There is a statistically significant difference, (*p* = 0.02), between the SLNB result and the MRI post-NAC result. 49.3% of the patients SLNB(−) present MPR in post-NAC MRI. 42.1% of patients SLNB(+) presented a MPR in the final MRI. 44.4% of non-migrations present mPR in the post-NAC MRI results, (Table 2).

Finally, there is a statistically significant difference (*p* = 0.02) between the SLNB result and LDN results. 64% of the patients SLNB(+) were positive LDN. 89% of the patients SLNB(−), were negative LDN, (Table 2).

## Discussion

Our study has been carried out for 10 years, in which the SLNB protocols for patients with LABC have been modified several times. As described above, we have a breast committee who agree on the diagnostic, prognostic and therapy criteria for all patients following the main guidelines as they are updated.

Our center makes pathological verification of the axillary lymph node positivity, and initially, a validation procedure for SLNB in LABC was performed, which consisted of performing the SLNB, with subsequent LDN in the same surgical procedure in patients with locally advance disease. Based on the SENTINA and ACOSOG studies, our protocol was modified, performing SLNB alone in patients with LABC with complete radiological response post-NAC. Posteriorly, we included the axillar clipping to the patients with clinic cN+. Those were the reasons because we have made the distinction between the different types of procedures. Currently, in patients with axillar involvement in post-NAC MRI, an axillary LDN is proposed like ACOSOG Z1071 study (Boughey et al. 2013).

Our study showed excellent IR results: SLNB IR for all the 114 patients was 92.10%; for patients with SLNB with LDN the IR was 89.8%; for patients with axillary clipping plus LDN and SLNB the IR was 91.6%; for patients with SLNB alone the IR was 100%; for patients with axillary clipping and SLNB alone the IR was 100%. These results could be attributed to the fact that the SLNB procedure is performed, in our center, with the dual method of radiotracer and blue dye and since 2017, we included the axillar clip too. Also, we obtained at least 2.13 sentinel nodes, that means that we were within the standard of ACOSOG recommendations (Boughey et al. 2013), and this allows to ensure us, (the surgeons and the nuclear physicians), to refine the extraction of the stained and radiolabeled node. The patients who underwent to SLNB alone, were patients with radiological CR, and all were SLNB(−), that means ypN0ypT0. The IR was 100%, which would justify its use in this special type of patients.

Non-migrations, in our opinion, do not depend on the technique, nor on the radiotracer, but depends on the pathological characteristics of the tumor, the infiltration degree and the response or not to chemotherapy. We found that 55% of the non-migrations were ypT1 post-NAC, 44% were ypN0 and 44.4% were mPR in the final MRI (*p* value of 0.017; < 0.001 and 0.020 respectively). These patients were treated with lymphadenectomy and 100% underwent RT.

3 of the SLNB(−) were positive LDNs, that means that they were false negatives. These patients were not CR to NAC, neither tumor nor lymph node, in both, post-NAC MRI and pathological results. These patients were of the triple negative and Luminal B, cN(+), which have a worse prognosis from the diagnosis. Many factors affect the result of the SLNB after NAC: large or bulky disease, fibrosis of the lymphatic drainage channel and cellular debris after NAC may block lymphatic vessels and divert, the mapping agents, to other non-sentinel lymph nodes (Hage et al. 2016; Tan et al. 2011). Chemotherapy induces both, in the tumor and in the axilla, marked fibrosis, foamy histiocytic infiltrate, calcifications, fat necrosis and hemosiderin deposition (Tan et al. 2011). We believe, like others (Hage et al. 2016; Tan et al. 2011), that the sentinel node probably responded to therapy, but the non-sentinel nodes and the rest of the tumor did not respond at all to NAC.

In the 114 patients of the general analysis, the FNR was 15.7%, very high and far from the usual reference standards which are between 8 and 10% (Krag et al. 2004). In patients who underwent to LDN and SLNB the FNR increased to 25%; but in patients with axillar clipping plus LDN and SLNB and in patients with axillar clipping and SLNB alone the FNR was reduced to 0%. We consider, like the previous literature recommend, that is justified use the clipping in patients cN+ confirmed by pathology.

36.2% of the negative LDN, were SLNB(+) in the group of axillar clip plus SLNB and LDN, which means that the SLN was the only positive node in these patients, and this indicates too, that our technique effectively could change the nodal staging even if the LDN is negative. These results were not a false positive result, because it was a valid pathologic result, in which we made the confirmation of positivity using the OSNA molecular method, which quantifies the expression of CK19, as a tumor marker, in the mRNA of the lymph node. This patient must be always treated like cN+.

Our study showed that axillar clipping and SLNB are complementary techniques, there were 15 patients (51.7%) in whom the clip was not found, that means more than a half. We consider that in these patients all the complementary techniques must be implement.

Our series demonstrate a correlation between the MRI and pathological results with a concordance in the CR of 65.6%. We also found an excellent nodal response to the NAC, with 51.19% of nodal response from cN+ to ypN0, better than other series that describe a complete nodal response of 40% (Hage et al. 2016). We believe that this was the result of the multidisciplinary teamwork of clinicals, radiologist, nuclear physicians, and pathologist, specialized in the treatment of this type of patients.

Our series demonstrate excellent survival results in the ten years of the study, we obtained an OS of 92%, with a DFS of 93%,

The OS for patients who underwent to SLNB alone, was 97% with a DFS of 96.8% with a follow-up of 93 months. The OS for patients who underwent SLNB and LDN was 89%, with a DFS of 89.8% with a follow-up of 120 months. The OS for patients who underwent to axillar clipping plus SLNB and LDN was 96% with a DFS of 95.8% with a follow-up of 80 months. That means that in patients ypT0 ypN0 who were submitted to SLNB alone and in the patients cN+ with axillar clipping plus LDN and SLNB, we obtained an OS > 95% and 100% of the patients with SLNB alone and axillar clipping survive. We consider that in the patients cN+ must be done axillar clipping, SLNB and LDN and again, we consider that patients ypT0ypN0 could be beneficiated with SLNB alone.

Mortality in our study was 7.01%. Most of these patients were of the intrinsic triple-negative subtype, followed by the LuminalB/HER2 subtype and those who did not undergo adjuvant treatment after surgery. Seven patients underwent to LDN, and one did not. This patient presented CR in post-NAC MRI result, and the patient was SLNB(−) and obtained ypT0ypN0 response and died due to progression of the contralateral locally advanced cancer.

Within the limitations of the study we find that the patients are already selected to NAC, so, there is no randomization. The total sample is small and therefore some groups are underrepresented. We also believe that a post-surgical verification of the presence of the axillary clip in lymphadenectomy should always be done via radiology, because unfortunately in many cases the clip was not found.

In the inferential analysis, the survival was lower, with statistical significance, (p-value < 0.05), for patients with triple negative and LuminalB/HER2 intrinsic subtype; with poor nodal response after NAC (ypN2-3); with progression or MPR in post-NAC MRI result and in patients with BRCA1/2 mutation. These findings are consistent with the literature and confirming the poor prognosis of patients with triple-negative intrinsic subtype and Her2, so a change of approach should be considered in these patients.

## Conclusions

- Our study presents excellent results of SLNB alone in LABC with complete nodal response with an OS and DFS > 95%.
- The FNR is very high in partial responders, so we cannot recommend the SLNB alone in LABC.
- We recommend, in cN+ patients con partial response in MRI post-NAC, axillar clipping, SLNB and LDN because in more than 50% of the patients with axillar clipping, this was not found, and because in 36% of the patients with negative LDN, the SLN obtained was the only positive node, so these techniques together decrease the FNR and improve the node staging, OS and DFS.
- This study is the first prospective study that assess OS and DFS in patients with LABC, all submitted to SLNB.

## Key points

**Question** What is the importance of survival and inferential analysis in patients with LABC submitted to SLNB?

**Pertinent findings** Our study presents excellent survival results in patients with LABC submitted to SLNB and with complete nodal response with an OS and DFS > 95%. We recommend, in cN + patients, axillar clipping, SLNB and LDN, these techniques decrease de FNR and increase the OS and DFS.

Axillar clipping and SLNB are complementary and always should be performed in LABC, because more than 50% of the patients with axillar clipping, this was not found, and because in 36% of the patients with negative LDN, the SLNB obtained the only positive node.

**Implications for patients care** This study showed that SLNB should be performed always in LABC treated with NAC because decrease the FNR and improve the node staging, OS and DFS.

## Data Availability

The datasets used and/or analyzed during the current study are available from the corresponding author on reasonable request.
